# Blood Plasma Quality Control by Plasma Glutathione Status

**DOI:** 10.3390/antiox10060864

**Published:** 2021-05-27

**Authors:** Tamara Tomin, Natalie Bordag, Elmar Zügner, Abdullah Al-Baghdadi, Maximilian Schinagl, Ruth Birner-Gruenberger, Matthias Schittmayer

**Affiliations:** 1 Institute of Chemical Technologies and Analytics, Faculty of Technical Chemistry, Technische Universität Wien, 1060 Vienna, Austria; tamara.tomin@tuwien.ac.at (T.T.); maximilian.schinagl@tuwien.ac.at (M.S.); 2 Center for Biomarker Research in Medicine, CBmed GmbH, 8010 Graz, Austria; natalie.bordag@lvr.lbg.ac.at (N.B.); abdullah.al-baghdadi@stud.medunigraz.at (A.A.-B.); 3 Translational Platform, Ludwig Boltzmann Institute for Lung Vascular Research, 8010 Graz, Austria; 4 Department of Dermatology and Venereology, Medical University of Graz, 8036 Graz, Austria; 5 Health Institute for Biomedicine and Health Sciences, Joanneum Research Forschungsgesellschaft mbH, 8010 Graz, Austria; Elmar.Zuegner@joanneum.at; 6 Division of Endocrinology and Diabetology, Department of Internal Medicine, Medical University of Graz, 8010 Graz, Austria; 7 Diagnostic and Research Institute of Pathology, Medical University of Graz, 8036 Graz, Austria

**Keywords:** glutathione, blood plasma, pre-analytical error, quality control, redox status, clinical chemistry

## Abstract

Timely centrifugation of blood for plasma preparation is a key step to ensure high plasma quality for analytics. Delays during preparation can significantly influence readouts of key clinical parameters. However, in a routine clinical environment, a strictly controlled timeline is often not feasible. The next best approach is to control for sample preparation delays by a marker that provides a readout of the time-dependent degradation of the sample. In this study, we explored the usefulness of glutathione status as potential marker of plasma preparation delay. As the concentration of glutathione in erythrocytes is at least two orders of magnitude higher than in plasma, even the slightest leakage of glutathione from the cells can be readily observed. Over the 3 h observation period employed in this study, we observed a linear increase of plasma concentrations of both reduced (GSH) and oxidized glutathione (GSSG). Artificial oxidation of GSH is prevented by rapid alkylation with N-ethylmaleimide directly in the blood sampling vessel as recently published. The observed relative leakage of GSH was significantly higher than that of GSSG. A direct comparison with plasma lactate dehydrogenase activity, a widely employed hemolysis marker, clearly demonstrated the superiority of our approach for quality control. Moreover, we show that the addition of the thiol alkylating reagent NEM directly to the blood tubes does not influence downstream analysis of other clinical parameters. In conclusion, we report that GSH gives an excellent readout of the duration of plasma preparation and the associated pre-analytical errors.

## 1. Introduction

In order to minimize pre-analytical variability, standard operating procedures (SOPs) for plasma and serum preparation have been continuously re-evaluated and discussed over the last decades [1]. Reasons for concern are that several factors involved in pre-analytical processing can be the critical determinants of the quality of obtained results. One such prominent variable is the pre-centrifugation delay, i.e., the lag time between blood collection and plasma preparation [2], which is shown to greatly influence the outcome of various analyses. According to a broad metabolite profiling study conducted by Kamlage et al. [3], a delay of 2 h at room temperature (RT) prior to plasma preparation significantly affected 22% of all monitored metabolites. In comparison, inducing hemolysis of grade 1 (passing the blood through a 25-gauge needle) affected only 18% of all metabolites included in the study [3]. Prominent changes in the metabolome were also reported by Bernini et al. [4], who demonstrated that the plasma metabolic profile was highly dependent on the time between blood collection and plasma preparation. In addition to impacting the metabolome, delays in plasma (or serum) preparation also affect the proteome. Both serum and plasma proteome profiles are significantly altered by processing delays [5]. Hsieh et al. showed that prolonged delays at RT led to a significant increase in the variation (coefficient of variation (CV) >20%) of 66% of all analytes [5]. While it is desirable to omit delays in plasma preparation altogether, this is often not feasible in clinical routine operation. To better assess the effects of prolonged incubation time between blood collection and plasma preparation, efforts have been made to discover potential indicators of blood processing delay. Lee et al. (2015) observed that if blood separation was postponed by 48 h, the plasma content of inorganic phosphate and potassium rose prominently [6]. Similarly, the same group also suggested a panel of cytokines (L-1β, GM-CSF, sCD40L, IL-8, MIP-1α, and MIP-1β) as potential indicators of the delay in plasma and serum preparations [7]. A comprehensive review of proposed preparation delay markers was published by Ruiz-Godoy et al. (2019) [2].

Nevertheless, there is a lack of a standardized, sensitive, stand-alone marker delivering a clear readout of the delay in plasma processing. Such a marker would have to be stable and would need to be detectable in plasma already after a 1 or 2 h processing wait, as those time points were shown to already significantly change analysis results [3,4]. In an attempt to tackle this conundrum, in the present study, we focused on the influence of plasma preparation delay on small molecular thiols with a focus on glutathione (GSH).

GSH is one of the most abundant intracellular thiols, and the ratio of GSH to oxidized glutathione (glutathione disulfide, GSSG) is often used as a readout of tissue oxidative state [8,9]. Addressing GSH and GSSG in biological fluids represents a challenge in itself, as GSH is prone to artificial oxidation during sample preparation [9,10]. However, if properly stabilized with a thiol-blocking (alkylating) agent at the point of sample collection, precise quantification can be ensured [9,10,11].

While treatment with alkylating reagents stabilizes the intracellular glutathione [9,10], the aim of this study was to investigate how a delay in blood separation of alkylated blood can influence the glutathione status of extracellular fluids, in particular plasma. To achieve this, we spiked blood collection tubes with N-ethylmaleimide (NEM), a potent thiol-blocking reagent [11,12], and incubated the blood for up to three hours at room temperature prior to centrifugation and plasma collection. Every hour (60 min), an aliquot of blood was taken and spun down to collect plasma, which was then used to monitor the concentration of GSH and GSSG as well as the GSH/GSSG ratio over time.

## 2. Materials and Methods

### 2.1. Study Design

The study was conducted in adherence to the Declaration of Helsinki and was reviewed by the ethical committee of the Medical University of Graz, Austria (31-116 ex 18/19, 16 January 2019). Prior to the commencement of any study activities, a written informed consent from all participants was obtained. The study protocol with the informed consent is available under https://www.drks.de DRKS-ID: DRKS00024807.

A study subgroup of 20 healthy volunteers (10 female, 10 male) was carefully selected to reduce biological and metabolic variation. Inclusion criteria were healthy Caucasian males or females, age from 20 to 30 years (mean 26 ± 3 SD), body mass index (BMI) from 18.5 to 25 kg/m^2^ (mean 22 ± 2 SD), abstinent of drug abuse for >1 year pre-trial, non-smoker or light smoker (≤1 cigarette/week), abstinent or light alcohol consumption (≤7 units/week, 1 unit = 10 mL or 8 g alcohol), overnight fasting (12 h), and training abstinence (24 h). Exclusion criteria included any acute or chronic diseases, hormonal contraception, medication with heparin (nonsteroidal or steroidal anti-inflammatory) in the last ten days, medication with antihistamines or selective serotonin reuptake inhibitors in the last four weeks, in vitro fertilization (IVF) treatment or any surgery in the last three months, any special diet form (e.g., vegan, gluten-free, malabsorption specific diets, ketogenic, etc.) or any other condition that would interfere with the safety of the participant, especially known anemia, blood or plasma donation within the last month, pregnancy, breastfeeding, intention of becoming pregnant or not using adequate contraception, mental incapacity, unwillingness or language barriers precluding adequate understanding or co-operation. All samples were collected within 3 weeks. Detailed characteristics of volunteers can be found in Supplementary Materials, Data 1.

### 2.2. Sample Collection and Plasma Preparation

Blood was collected in the morning between 8 and 10 a.m. (MESZ) with a 21 G butterfly needle, while sitting. Venipuncture was performed maximum once per arm. The tourniquet was released after 1 min, and blood collection was achieved in 4 to 9 min. Several tubes were collected (e.g., for clinical routine hematology or biobank storage); the first tube was discarded, and each tube was immediately inverted gently three times.

NEM stabilized sample collection and preparation. Shortly before the blood collection, VACUETTE^®^ (Grainer Bio-One, AT) blood collection tubes were spiked with N-ethylmaleimide (NEM, 75 mM) dissolved in 100 µL in phosphate saline buffer (PBS) to a final concentration of 2.5 mM in 3 mL K_3_EDTA tubes. After blood collection, tubes were kept upright at room temperature, and at regular intervals (0, 60, 120, and 180 min), 500 µL of NEM-stabilized blood was transferred from the blood collection container to a 1.5 mL microcentrifuge tube using a 1 mL trimmed pipet tip to minimize cell damage. Microcentrifuge tubes were then spun down for 10 min at 1300× *g* according to the standard operating procedure (SOP) [1] for plasma preparation, and 100 µL of plasma was carefully transferred to a new microcentrifuge tube and frozen till further analysis.

To analyze whether NEM affects common blood parameters, blood from 20 individuals was collected in VACUETTE^®^ (Grainer Bio-One, Kremsmünster, Austria) 8 mL Lithium Heparin Tubes spiked with either 100 µL of 200 mM NEM in PBS (2.5 mM final concentration) or 100 µL PBS as vehicle control shortly before sample collection.

### 2.3. Clinical Routine Hematology

Tubes for clinical routine hematology were transported (10–15 min) in isolated boxes, the temperature was logged and did not exceed the limits of 20–25 °C. The transport was within 2.5 h after phlebotomy, and hematology measurement was performed within 4 h at the Clinical Institute of Medical and Chemical Laboratory Diagnostics, Medical University of Graz. Tubes from the same volunteer were always handled similarly during transport and subsequent measurement. Hematological measurements were carried out on Sysmex Blood counters of XN or XE series (Sysmex Co., Kobe, Japan) by means of flow cytometry. All molecular analyses were carried out on a COBAS 8000 instrument (Roche Diagnostics, Rotkreuz, Switzerland) with the following methodologies applied: ions were measured through indirect potentiometry; alanine and aspartate aminotransferase, as well as glucose, creatinine, and cholesterol, were addressed spectrophotometrically; troponin T and thyroid-stimulating hormone via electro-chemiluminescence immunoassay (ECLIA) and C-reactive protein were measured with an immunological turbidity test.

### 2.4. Glutathione Measurements

Reduced (GSH) and oxidized glutathione (glutathione disulfide, GSSG) were addressed using a two-step alkylation protocol followed by liquid-chromatography coupled to mass spectrometry analysis (LC-MS/MS) as previously described [9] with a difference that the measurement was carried out on a TSQ Access Max triple quadrupole (Thermo Scientific, Waltham, MA, USA) operating in positive SRM mode. TSQ parameters were as follows: spray voltage 3000 V, capillary temperature 240 °C, vaporizer temperature 300 °C, and sheath gas, ion sweep gas, and aux gas pressures were 35, 0, and 5 units, respectively. Tube lens offset was set to 48 for the lower molar masses and skimmer offset was set to 0. The transitions with their corresponding collision energies are listed in Table 1.

### 2.5. Lactate Dehydrogenase (LDH) Activity Assay

The LDH activity of 14 plasma samples prepared after the indicated time delay (0, 60, 120, or 180 min after the blood collection) was addressed spectrophotometrically using a Lactate Dehydrogenase Activity Assay Kit (MAK066, Sigma Aldrich, St. Louis, MO, USA) according to the manufacturer’s instructions. Briefly, for each well, 25 µL of plasma was mixed with 25 µL of assay buffer and added to 50 µL of reaction mix (consisting of substrate mix diluted in the assay buffer). On each microtiter plate, duplicates of 3 µL of positive control were included. After the initial read, the plate was read continuously every five minutes for the total length of 20 min. Absorption values obtained after 10 min of incubation (per each time point) were all in the linear range and were selected for quantification.

### 2.6. Statistical Analysis

Data visualization and statistical analysis were performed with Microsoft Excel and R [13] (v4.0.2, packages plyr, stringr, readxl, ggplot2, dendsort, pheatmap, cellWise, Metaboanalyst R) [14,15]. The statistical significance of GSH or LDH activity was tested by applying one-way ANOVA or Student t-tests with a *p*-value of 0.05 as the significance threshold. If not stated otherwise, values are displayed as mean values ± standard error of mean (S.E.M).

For the analysis of NEM influence on clinical parameters, data distribution and scedasticity were investigated with Kolmogorov–Smirnov test and Brown–Forsythe Levene-type test, respectively, and multiple test adjustment by Benjamini–Hochberg (BH) (Supplementary Materials, Data 1). From the 26 parameters, three had to be excluded because the data behaved categorically (Baso, Eos, Mono; for abbreviations, see Table 2), and two had to be excluded due to too many missing values (below detection limit, CRP, TNTHS). All remaining 21 numeric parameters were found to be sufficiently normally distributed and homoscedastic for further statistical analysis without transformation.

Principal component analysis allowing for missing values and cellwise and row-wise outliers (R function MacroPCA) was performed centered and scaled to unit variance [16]. The number of components was set to cumulatively retain 80% of explained variance, here it was six. Orthogonal projections to latent structures discriminant analysis (OPLS-DA) was performed centered and scaled to unit variance (R function Normalization with scaleNorm = ”AutoNorm” and R function OPLSR.Anal) with a standard seven-fold cross-validation for the classification factor gender or NEM. Model stability was additionally verified with 1000 random label permutations. Detailed model results of MacroPCA and OPLS-DA, including scores, loadings, and S plot values, are given in Supplementary Materials, Data 1. Hierarchical clustering analysis was performed centered and scaled to unit variance (R function scale) per parameter. The dendrograms were clustered by Lance–Williams dissimilarity update with complete linkage (R function dist and hclust) and sorted (R function dendsort) at every merging point according to the average distance of subtrees and plotted at the corresponding heat maps (R function pheatmap).

## 3. Results

### 3.1. Glutathione Content in Plasma Increased with the Delay in Plasma Preparation

**Figure 1:**
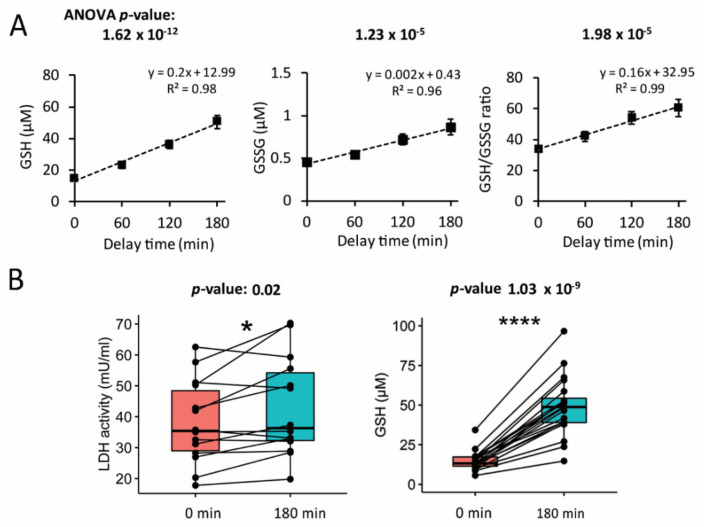
Concentrations of both GSH and GSSG increase over preparation delay time in plasma most likely due to hemolysis. (**A**) Absolute GSH concentration (left), GSSG concentration (middle), and GSH/GSSG ratio (right) content in the plasma of 20 volunteers after their blood was incubated for 0, 60, 120, and 180 min prior to plasma preparation. Points represent mean values (*n* = 19 or 20) ± S.E.M on which linear regression was applied. (**B**) Lactate dehydrogenase activity assay (left panel, *n* = 14) suggests an increase in LDH activity in plasma over time (after 3 h of incubation at RT, *: *p*-value < 0.05). In comparison, GSH concentration increased with a steeper slope over time, and the difference of the start and endpoint was considerably larger as reflected by the lower *p*-value (right panel, *n* = 20, ****: *p*-value < 10^−4^).

GSH is one of the most abundant intracellular antioxidants, with especially high concentration levels in red blood cells (in the range of 1.2–1.5 mM) [9,17]. As the concentration of GSH in the blood cells is orders of magnitude higher than its concentration in physiological fluids such as plasma or serum [8,9], even a slight “leakage” of glutathione from the blood cells can lead to a prominent increase in the GSH content of the liquid blood components. We detected a significant and strong increase of both GSH as well as GSSG in plasma by approximately 3.5 and 1.9 folds, respectively (Figure 1A), after a delay of plasma preparation at RT for up to 3 h. The increase in GSH content in plasma was continuously higher than the increase in GSSG, so that the GSH/GSSG ratio also rose steadily over time (Figure 1A). This increase of GSH plasma concentration was highly significant already after 1 h of preparation delay (paired Student’s *t*-test *p*-value = 2.34 × 10^−5^, Figure 1A, left panel). The same was true, although to a lesser extent, for GSSG content and GSH/GSSG ratio (paired Student’s *t*-test *p*-value (0 versus 60 min) for GSSG and GSH/GSSG: 1.24 × 10^−2^ and 1.73 × 10^−4^, respectively).

### 3.2. Increase in Glutathione Was Accompanied by Higher Lactate Dehydrogenase Activity of the Plasma

To be able to discriminate reduced glutathione leakage from active oxidized glutathione (GSSG) export by erythrocytes, we employed our recently published derivatization method based on stable-isotope-labeled N-ethylmaleimide [9]. This approach also prevents GSH loss due to oxidation. We hypothesized that the observed increase in glutathione content after the delays could indicate increased cellular damage. To test this hypothesis, we carried out a lactate dehydrogenase (LDH) assay, a common test for cells undergoing necrosis, apoptosis, and disruption of cellular membranes, resulting in the release of LDH [18]. Thus, in our setting, an increase in LDH in plasma could indicate leakage from blood cells. Comparing LDH activity of plasma prepared minutes after blood collection (0 min time point) to plasma collected after a delay of 3 h (180 min time point; in a pairwise manner, i.e., per volunteer), LDH activity of plasma was significantly higher (Student *t*-test *p*-value = 0.02) at the later time point (Figure 1B, left panel). However, pairwise comparison of individual GSH concentrations to LDH activities reveals higher significance of the GSH concentration (paired Student’s *t*-test *p*-value = 1.03 × 10^-9^), indicating far better sensitivity of GSH concentration as a marker of prolonged blood incubation time prior to plasma preparation (Figure 1B, right panel).

### 3.3. Common Blood Parameters Were Not Affected by NEM-Spiked Tubes

**Figure 2:**
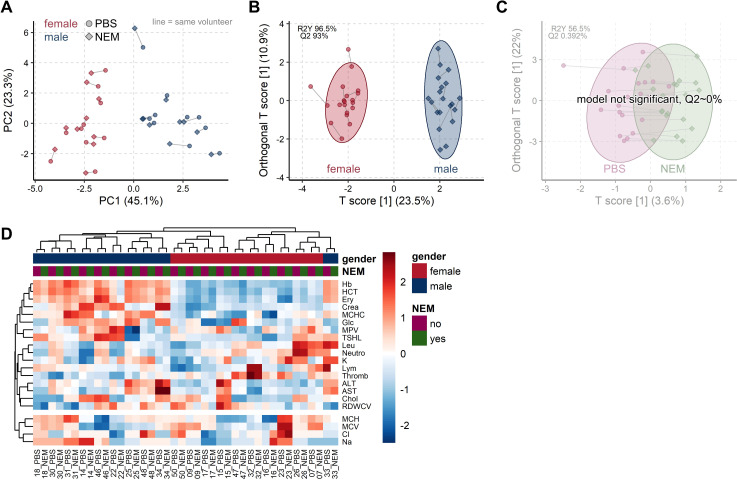
Addition of NEM to the blood tubes does not influence common clinical blood parameters. (**A**) The unsupervised PCA scores plot shows the very high similarity of all 21 clinical parameters for each of the 20 volunteers (connected by the gray line) when measured from tubes spiked with PBS (circles) or with NEM (diamonds). Samples are colored according to gender (female red, male blue), showing the expected strong group separation. (**B**) OPLS-DA scores plot reconfirm the PCA findings that clinical parameters significantly differ between gender, while (**C**) the addition of NEM to PBS has no significant impact ((**A,C**) values in Supplementary Materials, Data 1). (**D**) Heatmaps with hierarchical clustering show the similarity of obtained values between tubes with PBS or NEM, always clustering both samples from the same volunteer together.

**Figure 3:**
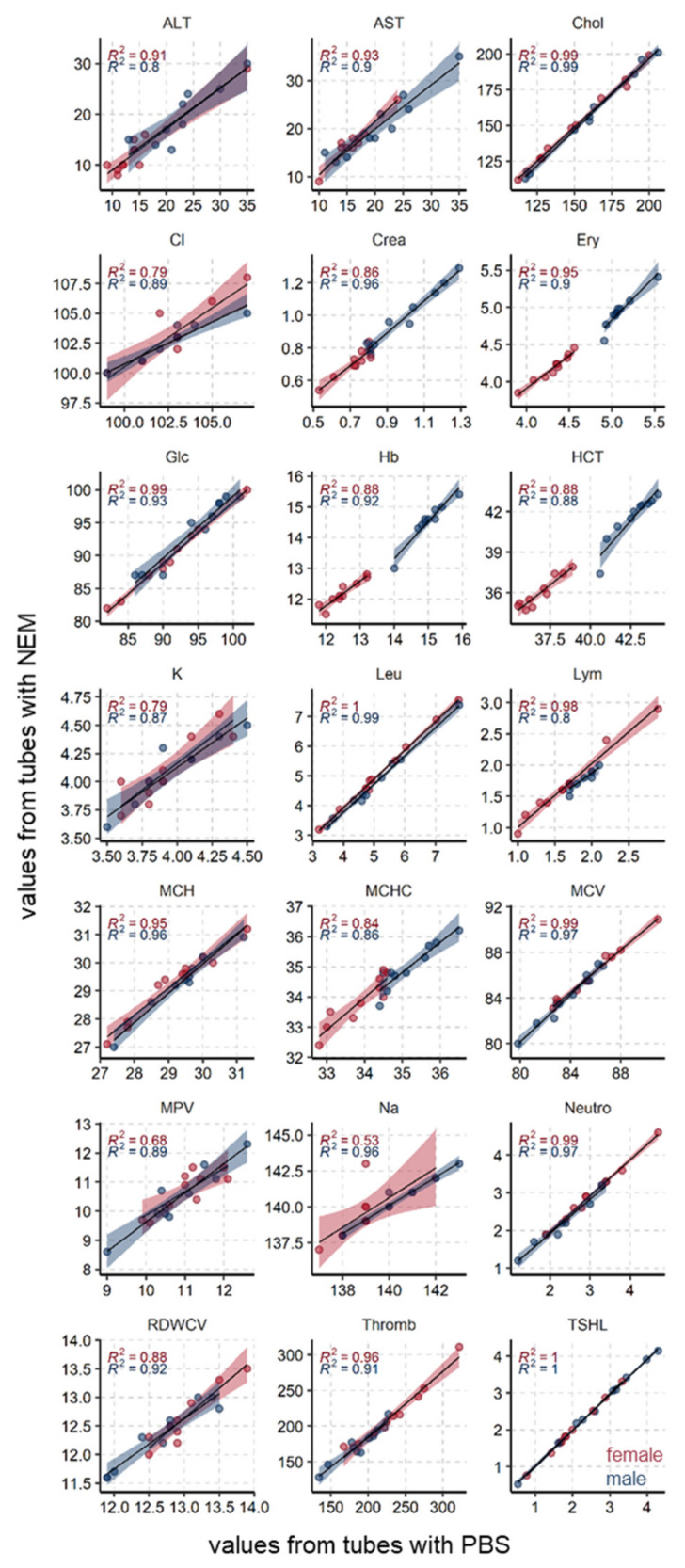
Direct comparison of clinical parameters measured from NEM- or PBS-spiked tubes. The linear correlation was calculated separately per gender, *R*^2^ values are given in blue for males and red for females, the corresponding transparent stripes mark the 95% confidence intervals.

As glutathione needs to be protected from artificial oxidation immediately upon sample collection [9,10], we next tested whether the addition of NEM directly to the blood tubes prior to blood collection would interfere with common downstream clinical applications. For that purpose, we compared clinical routine parameters covering hematology, electrolytes, and a number of biochemical compounds from blood collected to either NEM-containing (2.5 mM final concentration) or NEM-free (control; PBS used as a vehicle) Li-heparin blood collection tubes (Figure 2 and Figure 3; Table 2; and Supplementary Materials, Data 1).

Comparison of the cell counts for Baso, Eos, and Mono (for abbreviations see Table 2) was only qualitatively possible due to the categoric nature of the data. Most results were equal, if differences were found, these were within the expected measurement precision. The disease markers CRP and TNTHS were low as expected, confirming the excellent health status of the included volunteers. The values were often below the limit of detection leading to too many missing values for statistical analysis. Thus, from the 26 assessed clinical parameters, a total of 21 were available for statistical analysis.

We first employed the unsupervised multivariate method MacroPCA [16] to gain unbiased insight into the main driving factors. The scores plot shows that samples with NEM or PBS from the same volunteer were very near, hence very similar to each other (Figure 2A). Biological differences between females and males (factor gender) are well known and functioned here as a positive control. The gender differences are well visible in the scores plot by a strong group separation along the first component (*x*-axis), which reflects 45% of the variability in the dataset. Hierarchical clustering confirmed the dominant similarity between tubes from the same volunteer by always clustering these beside each other (Figure 2D). Overall, samples were almost perfectly clustered by gender, emphasizing that gender differences are much larger than minor deviations between tubes from the same volunteer. OPLS-DA confirmed that gender differences in clinical parameters are highly significant (Figure 2B) with a Q2 of 93% far above the cutoff, Q2 > 50%, while no significant differences were found between PBS- or NEM-spiked tubes (Figure 2C) with a Q2 of around 0%.

In addition, we performed a direct comparison by linear correlation. Values from NEM and PBS tubes correlated, in general, very well with each other for both genders (Figure 3). As expected, males had, in general, much higher red blood cell–related values (Ery, Hb, HCT, MCHC, MCV) and higher creatinine (crea) as a result of their higher muscle mass.

Furthermore, the majority of the parameters displayed a low mean within-subject coefficient of variation (CV_w_) of below 5% (Table 2). Among the parameters with a CV_w_ higher than 5% were alanine and aspartate aminotransferase (ALT and AST, CV_w_ = 11.7 and 5.7%, respectively), platelets (CV_w_ = 5.47%), and troponin (CV_w_ = 17.6%). A detailed list of the individual values of all measured parameters can be found in Supplementary Materials, Data 1. 

## 4. Discussion

Delay between blood collection and plasma preparation can introduce variability into consequent downstream analysis of the plasma metabolome [3,4] as well as proteome [5]. SOPs for plasma preparation suggest that blood centrifugation should be carried out within 4 h upon blood collection [19], a time period that was shown to be sufficient to shift plasma metabolomic signatures [4]. Nevertheless, the majority of studies investigating potential markers of the pre-analytical delay focused mainly on molecules that increase in plasma after or at the indicated 4 h mark [2], which, for many downstream analyses, might already be detrimental.

In contrast, here, we report NEM-stabilized GSH as potential early markers of plasma preparation delay. Adding NEM to phlebotomy tubes can easily be implemented during the manufacturing process by mixing NEM with anticoagulants before spray drying. Moreover, the reagent cost would be negligible—below EUR 0.02 per 7 mL tube. In contrast to unmodified GSH concentration, which would undergo a time-dependent decline due to oxidation, NEM-stabilized GSH accumulates in plasma over time. GSH concentration rose significantly already after 1 h of blood incubation at RT prior to centrifugation (0 versus 60 min time point paired Student *t*-test *p*-value of 1.16 × 10^−5^). Over the monitored period of 3 h, we observed that both GSH and GSSG content in plasma increased linearly (*R*^2^ = 0.98 and 0.96, respectively, Figure 1A) with longer centrifugation delay. However, as GSH increased faster than GSSG, this also led to a significant increase of the GSH/GSSG ratio in plasma. Therefore, both GSH and GSSG as well as the GSH/GSSG ratio constitute reliable indicators of delay prior to plasma preparation.

One potential reason for the observed increase in glutathione content in plasma could be time-dependent blood cell damage. It is known that incubation of blood in EDTA tubes at RT for a period of 24 h leads to approximately 2% hemolysis [20], which, given the drastically higher GSH content of blood cells compared to the cell-free blood fraction, could be more than enough to cause a noticeable rise of GSH content in plasma already within the first hours of blood collection. In support of this hypothesis, an LDH activity assay demonstrated that 3 h of blood incubation indeed resulted in increased cellular damage (Figure 1B, left panel). However, the time-dependent rise in LDH activity was significant only at the three-hour time point when tested in a pairwise manner (Student *t*-test *p*-value 0 vs. 180 min (LDH activity) = 0.02, Figure 1B), rendering it a far less sensitive marker than GSH (Student *t*-test *p*-value 0 vs. 180 min 1.03 × 10^-9^, Figure 1B).

Already, 0.3% of hemolyzed red blood cells result in a notable red coloring of blood-derived products due to hemoglobin release [21]. We did not observe any red coloring in plasma samples after 3 h RT incubation, but the increase in GSH corresponds to a release of ~4.3% of total erythrocyte GSH (at 0.42 erythrocyte volume fraction, 1.2 mmol/L GSH concentration). Therefore, we conclude, that processes apart from hemolysis also play a role in GSH release from the blood cells. While we cannot rule out lysis of non-erythrocyte cells as a source of GSH, multidrug-resistance-associated proteins have been reported to transport GSH across intact cellular membranes and could be a potential explanation for GSH increase in plasma [22].

The GSH concentrations in our cohort of 20 healthy volunteers were all found to be within a narrow range at any time point tested, making GSH a highly promising marker for plasma quality control. However, GSH levels of a larger and more diverse cohort, especially including patients with diseases known to perturb redox status, are required to define general applicable boundaries.

The second goal of this study was the incorporation of cysteine quenchers into the clinical routine, which would also enable precise redox status measurements. Clearly, the addition of NEM has to be considered when performing total antioxidant capacity assays as the results will not represent the major fraction of free thiols [23]. However, NEMylated thiols can simply be quantified by mass spectrometry, which adds an additional layer of information, and stabilization of thiols prevents systematic errors introduced by inevitable oxidation during plasma preparation [11]. In contrast, to measure total GSH after reduction of plasma, the selective measurement of NEM-stabilized free GSH avoids mixing of GSH from different pools such as mixed thiols and oxidized serum albumin. This not only simplifies data analysis but also ensures minimal starting GSH concentration and optimal assay sensitivity.

We also investigated whether spiking standard blood tubes with 2.5 mM NEM would show any influence on the most common routinely used blood parameters. The results from NEM-spiked tubes were found to be very similar to those of PBS-spiked tubes (as vehicle control) with several different statistical methods. The unsupervised methods MacroPCA and hierarchical clustering found a very high similarity of values from PBS and NEM tubes, while the expected large difference between males and females was as well detected (Figure 2). The significance was accordingly confirmed with the supervised machine learning method OPLS-DA. When directly comparing values from NEM to PBS tubes, a clear linear correlation existed with mostly high *R*^2^ > 0.9 (Figure 3). Lower *R*^2^ (0.58–0.8, e.g., in Na, Cl, K, MPV) can be rather attributed to the instrument’s precision and small range of values. The majority of the tested parameters (Table 2) showed very low paired coefficients of variation with most CV_w_ < 5%. The few exceptions were ALT (CV_w_ = 11.7%), AST (CV_w_ = 5.7%), and platelets (CV_w_ = 5.5%), which all showed *R*^2^ > 0.8. All of the listed CV_w_ values are in accordance with the previously published within-subject, biological variance range for each of the parameters. For example, according to the currently available databases on biological variation (BV) (version from 1999 [24] and 2014 update [25]), as well as more recent systematic review [26], the reported CV_w_ range for ALT is 11.1–58%, and 3.0–24% [25] or 32.3% for AST [26]. Regarding the platelets, the study from 2018 by Coskun et al. reported a mean CV_w_ value of 7.2% for platelets [27]. Values in this study were obtained from 30 individuals and corroborated the proposed CV_w_ ranges from the BV databases [24,25]. Altogether our results find no influence of NEM on the tested routine blood parameters.

## 5. Conclusions

**Figure 4:**
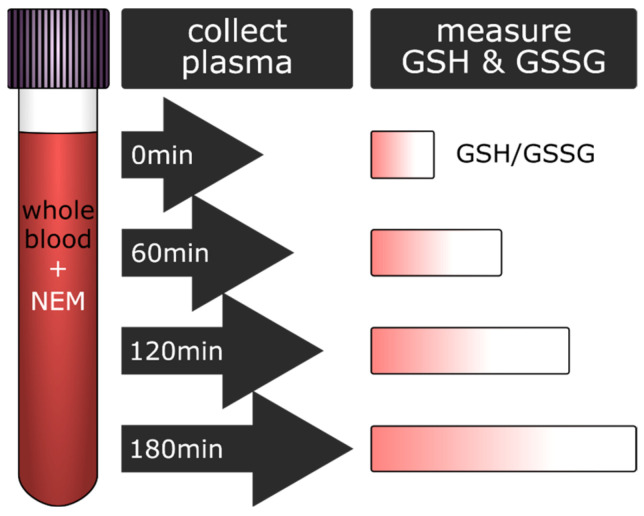
Summary of the workflow and the obtained results. Blood from 20 healthy volunteers was collected in pre-NEMylated blood collection tubes (2.5 mM final NEM concentration). At regular intervals (0, 60, 120, and 180 min), an aliquot of collected blood from each volunteer was spun down to collect the plasma, which was then used for further analysis and revealed an increase in GSH concentration in a time-dependent manner.

As a result of this study, we propose that GSH, GSSG, or the ratio of GSH/GSSG could be used as highly sensitive potential biomarkers for a centrifugation delay prior to plasma preparation. Plasma samples with a conspicuously high level of GSH could, therefore, be flagged, and other degradation-sensitive clinical parameters could then be evaluated in the light of a prolonged waiting period until preparation. The study workflow and the results are summarized in Figure 4.

In addition, we provide information regarding the rate of sample degradation of blood samples kept for up to three hours at RT. Lastly, in an attempt to aid the implementation of cysteine quenchers such as NEM into the standard clinical routine for precise downstream redox analyses, we demonstrate that addition of NEM to the blood tubes does not influence common routine clinical parameters. Overall, this study provides guidelines on how to track and consider pre-analytical variability, all in order to improve the confidence in the obtained results.

## 6. Patents

PCT/EP2019/072916

EP19758741.3

## Figures and Tables

**Table 1:** Multiple reaction monitoring (MRM) table of transitions for glutathione and glutathione derivatives. Transitions highlighted in bold and italic were used for quantitation.

Metabolite	Parent Ion	Product Ion	Collision Energy
***GSH***	***308***	***179***	***19***
***GSH-NEM (Transition 1)***	***433.16***	***201***	***22***
GSH-NEM (Transition 2)	433.16	304	15
***GSH-d5-NEM (Transition 1)***	***438.16***	***206***	***22***
GSH-d5-NEM (Transition 2)	438.16	309	15
***^13^*** ***C_2_, ^15^N-GSH-d5-NEM (IS)***	***441.16***	***206***	***22***
***GSSG***	***613.2***	***355.3***	***10***

**Table 2:** Detailed list of blood parameters from blood with PBS or NEM addition (mean ± SD). CV_w_ represents the mean of all standard deviations obtained per volunteer divided by the mean value of the given blood parameter. The transport occurred within 2.5 h after phlebotomy, and hematology measurement was performed within 4 h. Parameters that were not part of the statistical analysis are highlighted in italic.

Abbreviation	Full Name	Mean (PBS)	Mean (NEM)	CV_w_ (%)	Reference Range		Unit
					Male	Female	
**Ery**	erythrocytes	4.7 ± 0.4	4.6 ± 0.4	2.1	4.5–5.9	4.1–5.1	10^12^/L
**Leu**	leucocytes	5.1 ± 1.3	5.0 ± 1.2	2.6	4.4–11.3		10^9^/L
**Lym**	lymphocytes	1.8 ± 0.5	1.7 ± 0.6	3.1	1–4.8		10^9^/L
**Mono**	monocytes	0.3 ± 0.1	0.3 ± 0.1		*0.2*–*1.0*		*10^9^/L*
**Eos**	eosinophils	0.2 ± 0.2	0.2 ± 0.2		*0*–*0.7*		*10^9^/L*
**Baso**	basophils	0.0 ± 0.0	0.0 ± 0.0		*0*–*0.2*		*10^9^/L*
**Neutro**	neutrophils	2.8 ± 0.8	2.6 ± 1.0	2.8	1.8–7.7		10^9^/L
**Thromb**	platelets	210 ± 45	195 ± 41	5.5	140–440		10^9^/L
**ALT**	alanine amino transferase	18.5 ± 7.8	16.1 ± 6.7	11.7	0–45	0–35	U/L
**AST**	aspartate amino transferase	18.5 ± 5.9	19.0 ± 5.7	5.7	0–35	0–30	U/L
**Crea**	creatinine	0.9 ± 0.2	0.8 ± 0.2	2.3	0 - 1.2	0–1	mg/dL
**Cl**	chloride ions	103 ± 2	103 ± 2	0.4	95–110		mmol/L
**K**	potassium ions	4.0 ± 0.3	4.1 ± 0.3	2.6	3.5–5.0		mmol/L
**Na**	sodium ions	140 ± 2	140 ± 2	0.2	135	145	mmol/L
**Chol**	cholesterol	156 ± 30	154 ± 30	1.1	0–200		mg/dL
**Glc**	glucose	92.7 ± 5.6	91.8 ± 5.5	0.8	70–100		mg/dL
**Hb**	hemoglobin	13.8 ± 1.4	13.4 ± 1.3	2.1	13–17.5	12–15.3	g/dL
**HCT**	hematocrit	39.8 ± 3.2	38.8 ± 3.2	1.8	40–50	35–45	%
**MCH**	mean cell hemoglobin	29.3 ± 1.1	29.3 ± 1.1	0.5	28–33		pg
**MCHC**	mean corpuscular hemoglobin concentration	34.5 ± 1.0	34.4 ± 1.0	0.5	33–36		g/dL
**MCV**	mean corpuscular volume	84.7 ± 2.6	85.1 ± 2.5	0.3	80–98		fL
**MPV**	mean platelet volume	10.9 ± 0.8	10.6 ± 0.9	2.9	7–13		fL
**RDWCV**	Red blood cell distribution width	12.8 ± 0.5	12.5 ± 0.5	1.9	11–16		%
**TSHL**	thyroid - stimulating hormone	2.4 ± 1.0	2.3 ± 1.0	1.1	0.10–4.00		µU/mL

## Data Availability

The authors confirm that the data supporting the findings of this study are available within the article and its Supplementary Materials.

## Supplementary Materials

The following are available online at https://www.mdpi.com/article/10.3390/antiox10060864/s1, Supplementary Material, Data 1: Volunteer and clinical data, GSH, GSSG, LDH measurements, statistical results.
